# Effects of gestational and lactational exposure to low dose mercury chloride (HgCl_2_) on behaviour, learning and hearing thresholds in WAG/Rij rats

**DOI:** 10.17179/excli2016-315

**Published:** 2016-06-21

**Authors:** Deniz Sahin, Cem Onur Erdolu, Sabriye Karadenizli, Ahmet Kara, Gunce Bayrak, Sumeyye Beyaz, Buse Demir, Nurbay Ates

**Affiliations:** 1 Kocaeli University / Medical Faculty, Physiology, Kocaeli, Turkey; 2 Kocaeli University / Medical Faculty, Kocaeli, Turkey; 3 Sakarya University Training and Research Hospital, Otorhinolaryngology Department, Sakarya,Turkey

**Keywords:** inorganic mercury, passive avoidance, DPOAE test, Morris watermaze, hearing thresholds, WAG/Rij rats

## Abstract

We investigated the effects of inorganic mercury exposure during gestational/lactational periods on the behaviour, learning and hearing functions in a total of 32, 5-week-old and 5-month-old WAG/Rij rats (equally divided into 4 groups as 5-week and 5-month control mercury exposure groups). We evaluated the rats in terms of locomotor activity (LA), the Morris-water-maze (MWM) test and the passive avoidance (PA) test to quantify learning and memory performance; we used distortion product otoacoustic emission (DPOAE) tests to evaluate hearing ability. There were no significant differences between the 5-week-old rat groups in LA, and we detected a significant difference (p < 0.05) in the HgCl_2_-treated group in PA, MWM and DPOAE tests compared with the control group. The HgCl_2_-treated 5-week-old group exhibited worse emotional memory performance in PA, worse spatial learning and memory performances in MWM. There were no significant differences between the groups of 5-month-old rats in LA, MWM or PA. However, the DPOAE tests worsened in the mid- and high-frequency hearing thresholds. The HgCl_2_-treated 5-month-old group exhibited the most hearing loss of all groups. Our results convey that mercury exposure in young rats may worsen learning and memory performances as well as hearing at high-frequency levels. While there was no statistically significant difference in the behavior and learning tests in adult rats, the DPOAE test produced poorer results. Early detection of effects of mercury exposure provides medicals team with an opportunity to determinate treatment regimens and mitigate ototoxicity. DPOAE test can be used in clinical and experimental research investigating heavy metal ototoxicity.

## Abbreviations

HgCl_2_: inorganic mercury, LA: locomotor activity, MWM: Morris watermaze, PA: passive avoidance, DPOAE test: distortion product otoacoustic emission test 

## Introduction

Regular contact with heavy metals is encountered in various human activities, which is gradually exacerbated in terms of both professional and environmental exposure (Vassallo et al., 2011[42]; Mello-Carpes et al., 2013[30]). Among these heavy metals, concerns about exposure to mercury have increased, particularly within recent decades. Mercury is found in the environment in three species:

1. organic mercury (methylmercury, MeHg),
2. inorganic mercury (i.e., mercuric chloride, HgCl_2_) and
3. elemental or metallic mercury (Hg^0^).

Mercury is one of the most malignant and ubiquitous environmental toxins that causes a wide range of adverse health effects in humans. Due to its variety of forms, mercury is able to provoke countless effects (WHO, 1991[44]; Goyer, 1995[17]; Clarkson, 1997[11]; Peixoto et al., 2007[32]; Gao et al., 2008[15]).The effects of acute and chronic exposure to low mercury concentrations have been described for the kidneys, cardiovascular system, and neurological system (Azevedo et al., 2012[1]; Mello-Carpes et al., 2013[30]).

Inorganic mercury has been used for many years in medications, teething powders, skin creams and germicidal solutions. Mercury impacts the developing central nervous system (CNS), and induces subtle behavioral abnormalities, (learning deficits as well as memory deficits mostly, at dose levels below those related to obvious symptoms of neurotoxicity) (Newland et al., 2004[31]; Rasmussen and Newland, 2001[33]; Weiss et al., 2005[43]; Liu et al., 2009[28]). It has been reported that nervous system symptoms such as paraesthesia, fatigue, progressive weakness and neuropsychiatric disorders may be related to inorganic mercury exposure (Teixera et al., 2014[40]). In addition to that, organic mercury, the most common form of intoxication in humans, is progressively metabolized by intestinal microflora to inorganic mercury at a low rate per day (Bernhoft, 2012[3]; Teixera et al., 2014[40]). A human neonatal study performed by Gao et al. (2008[15]) revealed that increased prenatal methylmercury exposure was associated with decreased behaviour ability. This knowledge provides some support for the hypothesis that neurodevelopmental risks ensue from prenatal MeHg exposure resulting from fish consumption. 

Previous studies have shown that both acute and long-term exposure to mercury may result in alterations in both the peripheral and/or central auditory systems. Ionic and metabolic changes in the auditory system are believed to be responsible for this ototoxicity (Hoshino et al., 2012[19]). Until now, the significant majority of studies have focused on central and peripheral neurological paths of the auditory tract. However, there have been no adequate studies conducted about the sensorial part of the system. In this study, we focused on the sensorial part of the auditory system using distortion product otoacoustic emission (DPOAE) tests. 

WAG/Rij rats are preferred as an animal model for absence epilepsy in humans and they have many EEG and behavioural characteristics that are common with or similar to human absence epilepsy. The WAG/Rij strain of rats has been used for many studies of genetic epilepsy, colorectal cancer, and learning and memory performance (Hutteman et al., 2011[23]; Karson et al., 2015[25]).

We designed this experiment so that female rats were continuously given HgCl_2_ injections throughout their gestational and lactational periods to assess the risk to the developing brain from prolonged exposure to HgCl_2_. Therefore, we assumed that the dose level was low in adult rats in this experiment. The mothers continued to receive the injections after parturition, and thereby their offspring were exposed to HgCl_2_ through breast milk until weaning. We examined the effects on the CNS (or brains) of the offspring based on behavioural and DPOAE tests for possible cognitive dysfunctions, spatial learning, emotional memory performances and hearing loss when they reached 5 weeks and 5 months of age to observe age-dependency. 

## Materials and Methods

### Animals 

We obtained male and female WAG/Rij strained rats from Kocaeli University, Experimental Medical Research and Application Center (Kocaeli, Turkey). During the treatment, the rats had unlimited access to standard rat chow and tap water and they were kept at a density of two to three animals per cage at constant humidity, light cycle (12:12 h light-dark), and room temperature. Ethics approval was granted by the Kocaeli University Animal Research Ethics Committee (2014-11) (Kocaeli, Turkey). 

### Animal preparation

The female rats were mated individually with the male rats. Mating was verified via observation of spermatozoa in a vaginal smear, and this time was designated as Day 0 of pregnancy. Exposure was begun at pregnancy Day 0. The females were then placed in separate polycarbonate cages. The mothers received injections during their gestational and lactational periods.

Totally 32 offspring rats were equally divided into 4 groups as; 5 week-old control group, 5 week-old mercury group, 5 month-old control group, 5 month-old mercury group. Mean weights of the 5 week-old rat and 5 month old rats were 63.80 ± 1.3 g, 69.30 ± 2.6 g; 235.2 ± 6.8 g, 238.4 ± 12.3 g respectively.

### Mercury and treatments

Mercury (II) Chloride was purchased from Sigma-Aldrich M1136 (USA). The rats were divided into two groups: control (vehicle-saline solution im) and treated with mercury chloride during gestational and lactational periods (4.6 µg/kg as 1^st^ dose, 0.07 µg/kg/day as subsequent dose im, to cover daily loss). We made the injections between 9:00 am and 10:00 am, and doses were selected according to previous studies (Wiggers et al., 2008[45]; Szász et al., 2002[39]).

The dams were checked every morning for the presence of newborn offspring; when the pups were first observed was considered to be Postnatal Day 0 (PND 0). The pups remained with their mothers until they were weaned and given drinking water (PND 30). 

### Locomotor activity

A computerized animal activity monitoring system (Commat Ltd., Turkey) that is tracked and observed via an open field activity software package and comprises a Plexiglas chamber was used to assess locomotor activity (LA). The Plexiglas chamber (40 x 40 x 35 cm) had pairs of infrared photo beams and detectors assembled vertically every 4.5 cm and horizontally every 2.5 cm. The software program was used to detect and record the interruptions of the photocell beams. The total LA was expressed in terms of the sum of stereotypic, ambulatory, and vertical activities. We monitored the activity for 5 min continuously following acclimatisation to the test room illuminated for a period of one hour with two 36-Watt overhead fluorescent tubes with a light intensity of 18-45 lx on the cage floors and 190-220 lx in the centre of the room (Gocmez et al., 2015[16]).

### Passive avoidance

Animals learn to refrain from a formerly accustomed behaviour, namely, passing from the light chamber to the dark chamber, in the passive avoidance (PA) test (Komsuoglu Celikyurt et al., 2014[26]). The rats were tested in a step-through type PA apparatus (UgoBasile model 7551, Italy). The dimensions of the device were 22 x 21 x 22 cm and it had a guillotine door that separates the light and dark compartments of the device.

### Pre-acquisition trial

On the first day (the training trial), we placed the rats in the light compartment individually and allowed them to explore this light compartment. After 10 sec, the system automatically opened the door between the two compartments. At this stage, the animal was able to pass into the dark compartment freely.

### Acquisition trial

We conducted the acquisition trial 15 min following the pre-acquisition trial. We placed the rats in the light compartment. The system automatically opened the door dividing the two compartments after waiting for a 30-second-long adaptation period. The door automatically closed when a rat completely entered the dark compartment (four paws in) and for a period of 3 sec, an electric shock of 0.5 mA was applied on the rat's feet via the grid floor. We then took the animals from the dark compartment and placed them in their home cages. The system recorded the time that the animals took to enter the dark compartment. We discarded all the animals that failed to cross from the light compartment to the dark one within 300 sec from the experiment. We cleaned both compartments between each training session to eliminate olfactory cues.

### Retention trial

24 h after training, we returned animals to the light compartment and recorded their latency to enter the dark compartment (four paws in) to evaluate memory. We did not apply any footshocs in this trial. When a rat did not make its way entirely into the dark compartment within 300 sec, we returned it to its cage and recorded a maximum latency of 300 sec for that rat. The latency to enter the dark compartment was used as a criterion for retention performance of step-through avoidance behaviors.

### Morris Water-maze test

We used a circular pool (150 cm in diameter) that was filled with water at 25 °C as the water maze. To make the platform invisible, we placed small white pieces of plastic in the pool (Gocmez et al., 2015[16]). The pool was situated in a dimly lit soundproof test room along with various extra-maze visual cues.

The maze was separated into four quadrants, and three equally spaced points around the edge of the pool were utilized as starting positions. We varied the order of the release positions throughout the experiment. During the acquisition sessions, we placed an escape platform (10 cm in diameter) in one of the quadrants 1 cm below the water surface. For five daily sessions (3 trials per session), we trained the rats in the Morris water maze (MWM). We conducted all the five consecutive daily sessions between 9:00 am and 12:00 pm.

For each acquisition trial, we placed a rat in the pool from one of the randomly chosen three locations with its head facing toward the wall of the pool. The initiation of a trial was marked with the release of a rat into the pool. When a rat found the platform and climbed onto it, we terminated the trial and recorded the mean escape latency. The trial length was limited to a maximum of 60 sec. When a rat failed to climb onto the platform within 60 sec, we terminated the trial and the rat was guided to the platform by the experimenter using their hand.

The inter-trial interval was 30 sec. We left the rat on the escape platform before starting the next trial during the inter-trial interval. We placed the rat in the pool anew from a different location after the inter-trial interval, and upon its release, the next trial began. We returned the rat into its cage at the end of each session. We observed that as the animal learned the location of the hidden platform, escape latency typically declined during acquisition. In these cases, we recorded an escape latency of 60 sec.

We employed a 'probe trial' twenty-four hours following the final acquisition session to determine the rats' recollection of the location of the hidden platform. During this trial, we removed the platform from the maze and allowed each rat to search the pool for 60 sec. Within this period, we anticipated that the animals were to spend comparatively more time looking for the quadrant that previously contained the hidden platform compared to they would for the other three quadrants.

### Determination of distortion product otoacoustic emission (DPOAE)

The DPOAE test is a hearing assessment method that evaluates the outer hair cell function of the cochlea. With this technique, a wide frequency range of the cochlea can be evaluated by providing two different sound stimuli termed F1 and F2 to the external ear channel to obtain a frequency-specific response from the cochlea. High frequencies are more likely to suffer from ototoxicity because most ototoxic changes first affected the basal turn, or high-frequency region, of the cochlea before progressing to lower-frequency regions. In the current study, we studied frequencies between 4 and 10 kHz. 

The animals were anaesthetised via an intraperitoneal injection of xylazine (Rompun, Bayer Vital, Leverkusen, Germany) 5 mg/kg and ketamine (Ketalar, Eczacibasi Warner Lambert, Istanbul, Turkey) 50 mg/kg prior to the DPOAE measurements. We determined the depth of anaesthesia with the pedal reflex and to maintain anaesthesia and we administered a half dose of this initial cocktail as required. After an otoscopic examination to rule out possible middle-ear pathologies, we recorded DPOAE using the smallest probe in a silent room. We used a standard commercial MI 34 OAE apparatus cochlear emission analyser (MAICON Ltd., Berlin, Germany OTOsuite software) to elicit the DPOAEs from each ear of the experimental animals. The cubic difference distortion products (2F_1_ - F_2_) were performed in the General Diagnostic mode, and the F1/F2 frequency ratio was set to be 1.22 to obtain the most powerful responses. We performed the measurements at 4004, 4502, 5000, 5596, 6299, 7099, 7993, 9004 and 10000 Hz F_2_frequencies at 6 points per octave.

## Results

### Locomotoractivity

The LA results of all of the experimental groups are listed in Table 1(Tab. 1). The total activity was not significantly decreased in both the 5-week-old and 5-month-old HgCl_2_-exposed groups compared with their own control groups (1202.0 ± 85.9, 719.9 ± 6.4, 1463.0 ± 123.6 and 1003.0 ± 108.8, respectively). Moreover, the HgCl_2_-exposed 5-month-old rats exhibited the least total activity of all of the groups. We compared to a same age-matched control group as an HgCl_2_-exposed group and a control group.

### Passive avoidance test 

Latency to enter the dark compartment was recorded for the HgCl_2_-exposed 5-week-old rats, the HgCl_2_-exposed 5-month-old rats, the 5-week-old rats and the 5-month-old WAG/Rij rats (25.29 ± 5.39, 76.64 ± 35.47, 89.66 ± 30.95 and 101.30 ± 32.29 sec, respectively). Latency to enter the dark compartment was decreased in both the HgCl_2_-exposed groups (5-week-old and 5-month-old) compared with their own control groups. 

**Figure 1:**
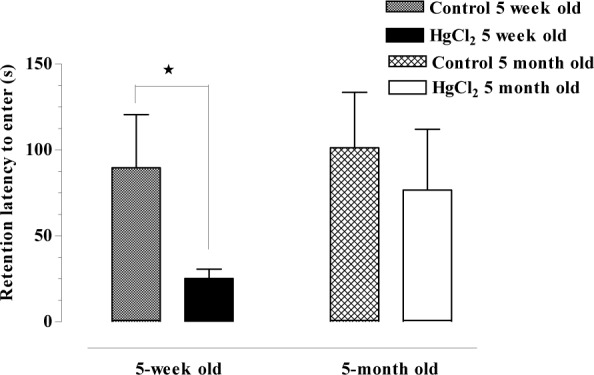
Latency to enter the dark compartment of HgCl_2_-5-week-old, HgCl_2_-5-month-old; 5-week-old, 5-month-old WAG/Rij rats (*p < 0.05, ANOVA: Kruskal-Wallis test; post test: Dunns: Compare selected pairs of columns)

Latency to enter the dark compartment was present in both the 5-week-old (p < 0.05) and 5-month-old (p > 0.05) groups. The HgCl_2_-exposed rats exhibited a larger decrease than their own control groups (Figure 1(Fig. 1)).

### Morris water maze

**Figure 2:**
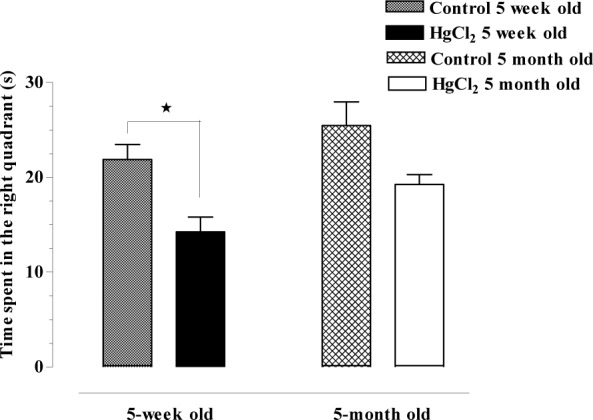
Time spent in right quadrant of HgCl_2_-5-week-old, HgCl_2_-5-month-old; 5-week-old, 5-month-old WAG/Rij rats (*p < 0.05, ANOVA: Kruskal-Wallis test; post test: Dunns: Compare selected pairs of columns)

The time spent in the right quadrant was decreased in both the 5-week-old and 5-month-old HgCl_2_-exposed groups compared with their own control groups (14.25 ± 1.56, 19.26 ± 1.03, 21.88 ± 1.59 and 25.47 ± 2.47 sec, respectively). The time spent in the right quadrant in both the 5-week-old (p < 0.05) and 5-month-old (p > 0.05) HgCl_2_-exposed rats was more decreased than these groups' own control groups (Figure 2(Fig. 2)). 

### DPOAE test results

When we analyzed the mean DPOAE thresholds, we noted that the 5-week-old and 5-month-old rats in the control group exhibited better test results for all of the frequencies than the HgCl_2_-exposed 5-week-old and HgCl_2_-exposed 5-month-old rat groups. However, only the differences at 10000, 9004, and 7993 Hz for the 5-week-old rats and the difference at 7099 Hz for 5-month-old rats were statistically significant (one-way ANOVA, F = 3.510, p = 0.024, Table 2(Tab. 2)). Intra-group analyses of the 5-week-old rats' and 5-month-old rats' test results revealed that both of the groups had age-related hearing loss at all frequencies. However, statistically significant differences were greater in the HgCl_2_-exposed group, particularly for the middle and higher frequencies (one-way ANOVA, F = 3.510, p = 0.024, Table 2(Tab. 2)) (7099, 6299, 5596, 5000 and 4004 Hz for the HgCl_2_ group and only 4004 Hz for the WAG/Rij group (Table 2(Tab. 2)).

## Discussion

We describe, for the first time, the age-dependent effects of gestational and lactational chronic, low-dose inorganic mercury exposure on long-term behaviour, memory and hearing in young adult and adult WAG/Rij rats. These animals constitute a valid model of genetic absence epilepsy and depressive-like behaviour. This study was designed to verify the effects of long-term exposure to low inorganic mercury doses on behavioural in young adult and adult life; and to make estimation of the common human professional exposition was one of the objectives of our study. The model for long-term mercury exposure was previously established in rats (Wiggers et al., 2008[45]) and as is the case with occupational exposure, is attained by low-dose exposure to mercury chloride (Szász et al., 2002[39]).

We have shown that chronic exposure to low-dose mercury chloride impairs performance of different memory types, general behaviour and hearing thresholds in the early life of 5-week-old WAG/Rij rats that were exposed mercury chloride during their gestational and lactational periods. The rats in the HgCl_2_-exposed group exhibited no different emotional and spatial memory performances on either the PA or MWM tests compared with their age-matched controls at 5 months. Day et al. (2005[12]) proposes that the age-dependent effects of mercury that compensatory mechanisms are compromised when dually burdened by methylmercury and aging processes. According to our results, although the age-dependent effects of mercury chloride seem to be reversible or tolerable in 5-month-old WAG/Rij rats, the DPOAE test revealed that there were seriously progressive deleterious effects of mercury chloride in exposed rats beyond all of the behavioural tests.

One of the inorganic forms of mercury is mercury chloride. While mercury is most commonly seen in inorganic form (Rosales et al., 2005[34]), the majority of research focused on intoxication effects of mercury in CNS have included others forms, for example, organic (Castoldi et al., 2001[7]; Fujimura et al., 2012[14]; Maia et al., 2009[29]) or vapour states (Yoshida et al., 2011[49]; Gul et al., 2012[18]; Yasutake et al., 2012[48]). All studies have found that this substance is extremely toxic to organisms. It has been argued in previous literature that before absorption, it is possible for inorganic mercury to methylate in the gut lumen (Rudd et al., 1980[35]) and cross the placental barrier (Yang et al., 1996[47]; Chehimi et al., 2012[8]). Accordingly, inorganic mercury can act at CNS, and mercury is especially damaging for the development of the brain (Azevedo et al., 2012[1]; Trasande et al., 2005[41]). Several researchers have underlined that mercury exposure during pregnancy (Cagiano et al., 1990[6]), the neonatal period (Yoshida et al., 2011[49]), and young life (Freire et al., 2010[13]) can lead to negative consequences and may result in brain functioning deficits in the adulthood period. According to Liang et al. (2003[27]), the developing CNS is more sensitive to damage from mercury than the adult CNS. Our results affirm that lower concentrations of HgCl_2 _induce behavioural, cognitive impairment and hearing-threshold deficits in 5-week-old rats and progressive hearing thresholds deficits in 5-month-old rats.

Zanoli et al. (1994[50]) showed that a single dose (8 mg/kg) of methyl mercury administered on the 15^th ^gestational day in rats had negative impact on the PA task when the animals were 8 weeks old, which shows that exposure during a crucial period of development of the CNS to methyl mercury leads to learning and memory deficits. Exposure to mercury in different forms may provoke diverse impacts on the CNS. In this sense, our current findings suggest that chronic HgCl_2_-treated animals exhibited worse emotional and spatial memory performances on the PA and MWM tests compared with their age-matched controls at 5 weeks. However, we found no significant alterations in the LA test. Non-significant LA results do not imply that mercury does not have detrimental and toxic effects. Brookes and Kristt (1989[5]) and Choi and Kim (1984[10]) demonstrated that HgCl_2_ concentrations under 1 μg/g are not toxic to *in vitro *cultured CNS tissues. Yoshida et al. (2011[49]) detected noteworthy alterations in gene expression in brains of mice contacted with low levels of mercury vapour during their postnatal development, in spite of the fact that their ability to learn was not affected. Hu et al. (2012[20]) administered rice contaminated with mercury to 3-week-old rats and measured in MWM that this procedure negatively affected spatial learning and memory of the rats. When it comes to effects of mercury on human beings, the effects of mercury exposure on cognitive functions in adulthood have only been reported in a few studies, a great deal of which focused on the results of uncontrolled exposure of humans to mercury (Smith et al., 1983[38]; Sletvold et al., 2012[37]) or in rats, through the course of a short exposition (Hu et al., 2012[20]). The long-term outcomes of chronic low-dose mercury chloride exposure (regional, occupational, etc.) are less well understood, and clinical studies on this topic are very limited.

Xu et al. (2012[46]) found that inorganic mercuric chloride (HgCl_2 _at 0.025-25 μM) not only caused neuronal degeneration but also perturbed neuronal excitability. Basu et al. (2007[2]) found decreases in NMDA receptor levels in the basal ganglia, cerebellum, brain stem and occipital cortex that are dependent on mercury concentration. Indicating that mercury can significantly reduce NMDA receptor levels even at dietary concentrations as low as 0.1 ppm, these findings raise both ecological and physiological concerns (Sanchez, 2015[36]).

AMPA receptor (AMPAR) membrane trafficking regulation is critical with regards to synaptic plasticity as is the case for learning and memory (Zhang, 2015[51]). The activation of NMDA receptors may contribute to better spatial and emotional memory performances. This situation may explain the deterioration in the MWM and PA test results of 5-week-old rats exposed to mercury.

Furthermore, gradually decreased levels of mercury and the ameliorating neuronal development of young rats after the end of inorganic mercury exposure might contribute to normalising cognitive and spatial functions in 5-month-old rats because the behavioural test results of 5-month-old rats were not statistically significant.

On the other hand, our results showed that there are seriously progressive effects of gestational and lactational inorganic mercury exposure. Beyond all of the behavioural tests we conducted, these seriously progressive effects may be disclosed by the DPOAE test.

The ototoxic effect of mercury has been studied previously using objective and subjective test methods; the cochleotoxic effect of mercury is a less-studied topic. We determined the cochleotoxicity using DPOAE tests. Otoacoustic emissions can be suppressed in part centrally through the superior olivary complex. Lateral and medial olivocochlear bundles' axons project from the superior olive and leave the brainstem as a ventral component to the inferior vestibular nerve. As the Oort's vestibulocochlear anastomosis, they join the cochlear nerve. Axons of the lateral olivocochlear bundle synapses with afferent neurons from the cochlea. Axons of the medial olivocochlear bundle terminate at the base of cell bodies of the outer hair cells. It is generally believed that the medial efferents counteract the amplifying effects of the outer hair cells. This effect is probably mediated by acetylcholine (Bolay et al., 2008[4]).

Many studies have reported that various insults, such as mercury toxicities and chemicals, induce neurological disorders or hearing loss in vivo and that those disorders correspond to changes in the Na^+^/K^+^-ATPase activities of the brain regions and cochlear lateral wall (Huang et al., 2007[21]; Cheng et al., 2005[9]).

Igarashi et al. (1992[24]) carried out a histochemical experiment, in which it was understood that mercury deposits usually concentrate in the cerebellum and in parts of the vestibular nerves, spiral ganglion, striavascularis, and cochlear nerves. In this experiment, there was a small mercury accumulation in the acoustic maculae and in the cochlea in the vestibule; and it was found that there was only a single instance of mercury accumulation in the organ of Corti. Our results were consistent with the results of Igarashi et al. (1992[24]).

In terms of the results of the 5-week-old rat groups, we noted that the HgCl_2_ group exhibited poorer test results for all frequencies compared with the control group. The differences were statistically significant at higher frequencies. These results imply that exposure to inorganic mercury during gestational and lactation periods results in ototoxicity; this toxicity can be monitored using the DPOAE test, which is an easy and objective test method. Additionally, in the 5-month-old rats, both groups exhibited age-related changes. However, our results demonstrated that the HgCl_2_-exposed group was more affected by hearing thresholds than the control group. Moreover, the largest hearing loss was recorded in the 5-month-old HgCl_2_ group. This result can be interpreted as age-related hearing loss being more deteriorated by HgCl_2_ exposure. Our results indicate that the DPOAE test is more sensitive than the other tests used in this study to reveal inorganic mercury toxicity, particularly for late effects in aged rats.

According to Huang et al. (2011[22]), absorption in gastrointestinal tract and accumulation of mercurial compounds in the brainstem are responsible for auditory dysfunction. The findings reached by Huang et al. (2011[22]) show that exposure to low-dose mercurial compounds (the probable concentrations for human exposure) in perinatal stages can induce irreversible ototoxicity; additional contact may end in severer toxic effects in future developmental stages. According to Liang et al. (2003[27]), mercury has targeted the sensory cochlea and vestibular apparatus of the inner ear containing the central auditory structures and hair cells. In addition to that, it has been shown by neurobiochemical studies of brainstem tissues that ototoxicity triggered by mercurial compounds could be mediated by oxidative stress damage, altered Na^+^/K^+^-ATPase activities and NOx levels of the brain regions and the cochlear lateral wall. This explanation may be the most appropriate to interpret our results. 

Additional studies are necessary to better understand the exact pathophysiological mechanism of long-term mercury exposure and its neurotoxicity. For this purpose, cochlear dissection and brain slices of rats must be obtained and analyzed using immunohistochemistry techniques. Moreover, given the urbanization and industrialization of today's rapidly growing world, it is important to raise public awareness about mercury and limit exposure to mercury as a public health priority.

## Conclusions

We have shown that chronic exposure to low-dose mercury chloride impairs the performance of different memory types and hearing in WAG/Rij rats that were exposed to mercury chloride during their gestational and lactational periods. Although age-dependent effects of mercury chloride appear to be reversible or tolerable by the behavioural tests, DPOAE test results revealed that there were seriously progressive deleterious effects of mercury chloride in rats exposed to mercury. Medical teams may find a chance to identify treatment methods and alleviate hearing changes with the early diagnosis the effects of mercury exposure. In this sense, in experimental and clinical studies that research hearing loss and heavy metal ototoxicity may benefit from DPOAE tests. However, we do not know the exact mechanisms involved in these effects; additional research is accordingly necessary. However, our results are important because information about age-dependent mercury chloride exposure with long-term outcomes on both animal models and humans is very limited in the literature.

## Acknowledgements

We thank Si-Ser Hearing Centers for providing us hearing threshold assessment equipment for DPOAE tests.

## Disclosure

The authors declare that there are no conflicts of interest. 

## Figures and Tables

**Table 1:**
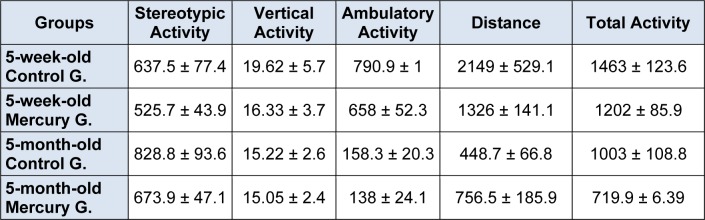
Effect of chronic mercury treatment on the locomotor activities of the rats (own age matched between Hg vs control groups, p > 0.05 )

**Table 2:**
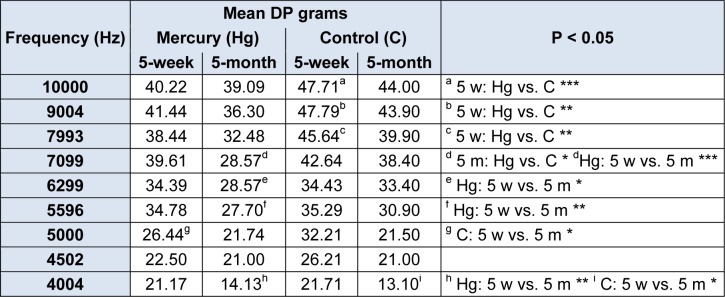
DPOAEs of HgCl_2_-5-week-old, HgCl_2_-5-month-old; 5-week-old, 5-month-old WAG/Rij rats. (5 w = 5-week, 5 m = 5-month, vs = versus)
